# Draft Genomic Sequences of Chromobacterium sp. nov. Strains MWU13-2610 and MWU14-2602, Isolated from Wild Cranberry Bogs in Massachusetts

**DOI:** 10.1128/genomeA.00332-18

**Published:** 2018-04-12

**Authors:** Kory O’Hara-Hanley, Alisha Harrison, Scott D. Soby

**Affiliations:** a Biomedical Sciences Program, College of Health Sciences, Midwestern University, Glendale, Arizona, USA; b College of Veterinary Medicine, Midwestern University, Glendale, Arizona, USA

## Abstract

Chromobacterium sp. nov. strains MWU13-2610 and MWU14-2602 were isolated from cranberry bogs in the Cape Cod National Seashore. These nonpigmented bacteria represent two new presumptive species of the rapidly growing genus Chromobacterium. Gene homologs are present for multiple antibiotic resistance, virulence functions, and prophages.

## GENOME ANNOUNCEMENT

Chromobacterium sp. nov. strain MWU13-2610 and Chromobacterium sp. nov. strain MWU14-2602 were isolated from native cranberry bogs at Race Point and High Head at the Cape Cod National Seashore in Massachusetts. These bacteria represent two new presumptive species of the rapidly growing genus Chromobacterium (1–9). The genomes were sequenced at the Arizona State University College of Liberal Arts and Sciences (CLAS) Genomics Core facility using an Illumina MiSeq platform. Genomic DNA was sheared to approximately 600-bp fragments using the Covaris M220 ultrasonicator, and Illumina libraries were generated on an Apollo 384 liquid handler (Wafergen) using the Kapa Biosystems library preparation kit (catalog number KK8201). DNA fragments were end repaired and A tailed, as described in the Kapa protocol. Combined indexes/adapters (catalog number 520999; Bioo) were ligated onto each sample and multiplexed into one lane. Adapter-ligated molecules were cleaned using AMPure beads (catalog number A63883; Agencourt Bioscience/Beckman Coulter) and amplified with a Kapa HiFi enzyme. Libraries were analyzed on an Agilent Bioanalyzer and quantified by quantitative PCR (qPCR) (catalog number KK4835; Kapa library quantification kit) before multiplex pooling and sequencing in a 2 × 300-bp paired-end (PE) flow cell on the MiSeq platform (Illumina). Adapters were computationally segregated and trimmed in the Illumina BaseSpace pipeline and then partially assembled using the Velvet assembly tool (10). The Chromobacterium sp. nov. MWU13-2610 genome has 62.4% GC content and consists of 4,387,023 bp distributed over 130 scaffolds, 77 of which are larger than 1 kbp. The largest contig is 445,097 bp, the *N*_50_ value is 105,364 bp, and the *N*_75_ value is 76,063 bp, with a sequence coverage of 51.4×. The Chromobacterium sp. nov. MWU14-2602 genome has 63.36% GC content and consists of 4,532,842 bp distributed over 233 scaffolds, 149 of which are larger than 1 kbp. The largest contig is 401,085 bp, the *N*_50_ value is 69,582 bp, and the *N*_75_ value is 32,238 bp, with a sequence coverage of 60.3×. These two genome sequences were compared using the Genome-to-Genome Distance Calculator (GGDC) provided online by the DSMZ (11). Chromobacterium sp. nov. MWU13-2610 and Chromobacterium sp. nov. MWU14-2602 were 30.2% homologous, indicating that they belong to different species.

*Ab initio* gene prediction was performed on the assembly using PATRIC (https://www.patricbrc.org) (12). The genome of MWU13-2610 contains 4,360 predicted genes, of which 3,055 were predicted to have functional assignments. The genome has multiple potential antibiotic resistance genes, including those for β-lactamases, catalase (*katG*), drug efflux pumps, and aminoglycoside transferases. Although there is no indication that MWU13-2610 is pathogenic, it may contain virulence genes that encode type III secretion systems and the chaperone protein SicA. The genome of MWU14-2602 contains 4,448 predicted genes, of which 3,139 have predicted functions. It contains multiple potential antibiotic resistance genes, including those encoding catalases, drug efflux pumps, and aminoglycoside transferases. Both genomes were scanned for prophages using PHAST (13), and both contain prophage genes. MWU13-2610 has three complete prophages, and MWU14-2602 has one complete prophage genome.

### Accession number(s).

This whole-genome shotgun project has been deposited at DDBJ/EMBL/GenBank under the accession numbers PPTF00000000 (MWU13-2610) and PQWB00000000 (MWU14-2602). The versions described in this paper are PPTF01000000 and PQWB01000000, respectively.
